# REEP6 deficiency leads to retinal degeneration through disruption of ER homeostasis and protein trafficking

**DOI:** 10.1093/hmg/ddx149

**Published:** 2017-05-05

**Authors:** Smriti A. Agrawal, Thomas Burgoyne, Aiden Eblimit, James Bellingham, David A. Parfitt, Amelia Lane, Ralph Nichols, Chinwe Asomugha, Matthew J. Hayes, Peter M. Munro, Mingchu Xu, Keqing Wang, Clare E. Futter, Yumei Li, Rui Chen, Michael E. Cheetham

**Affiliations:** 1 Department of Molecular and Human Genetics; 2 Human Genome Sequencing Center, Baylor College of Medicine, Houston, TX 77030-3411, USA; 3 UCL Institute of Ophthalmology, 11-43 Bath Street, London EC1V 9EL, UK; 4 Department of Ophthalmology; 5 Department of Molecular Physiology and Biophysics, Baylor College of Medicine, Houston, TX 77030-3411, USA

## Abstract

Retinitis pigmentosa (RP) is the most common form of inherited retinal dystrophy. We recently identified mutations in *REEP6*, which encodes the receptor expression enhancing protein 6, in several families with autosomal recessive RP. REEP6 is related to the REEP and Yop1p family of ER shaping proteins and potential receptor accessory proteins, but the role of REEP6 in the retina is unknown. Here we characterize the disease mechanisms associated with loss of REEP6 function using a *Reep6* knockout mouse generated by CRISPR/Cas9 gene editing. In control mice REEP6 was localized to the inner segment and outer plexiform layer of rod photoreceptors. The *Reep6^-/-^* mice exhibited progressive photoreceptor degeneration from P20 onwards. Ultrastructural analyses at P20 by transmission electron microscopy and 3View serial block face scanning EM revealed an expansion of the distal ER in the *Reep6^-/-^* rods and an increase in their number of mitochondria. Electroretinograms revealed photoreceptor dysfunction preceded degeneration, suggesting potential defects in phototransduction. There was no effect on the traffic of rhodopsin, Rom1 or peripherin/rds; however, the retinal guanylate cyclases GC1 and GC2 were severely affected in the *Reep6* knockout animals, with almost undetectable expression. These changes correlated with an increase in C/EBP homologous protein (CHOP) expression and the activation of caspase 12, suggesting that ER stress contributes to cell death. Collectively, these data suggest that REEP6 plays an essential role in maintaining cGMP homeostasis though facilitating the stability and/or trafficking of guanylate cyclases and maintaining ER and mitochondrial homeostasis.

## Introduction

Retinitis pigmentosa (RP, MIM 268000) is a genetically heterogeneous disorder resulting in loss of photoreceptor cells in the retina, the light sensitive tissue located in the posterior region of the eye. The genes with RP associated mutations encode proteins involved in a diverse range of pathways that are essential for photoreceptor structure, function, homeostasis, and survival (1–4). This heterogeneity is a reflection of the complexity of photoreceptor cell biology and function. Rod photoreceptors are specialized highly sensitive light sensing neurons that mediate peripheral and night vision. Amongst their specializations are the rod inner segment, which concentrates biosynthetic and metabolism related cellular organelles, such as the endoplasmic reticulum (ER) and mitochondria, and a highly specialized cilium the outer segment (OS), which contains all the visual transduction components essential for light to be converted into electrical impulses that are transmitted through the retina and to the brain via retinal ganglion cells. (5–7). This process is referred to as phototransduction, and it is an essential process to initiate vision.

Phototransduction proteins are produced in the inner segment and translocated to the rod OS by intracellular protein transport and vesicular trafficking (5,8). Protein trafficking in photoreceptors is critically important to maintain the structure, function, and overall photoreceptor homeostasis. Aberrant protein function and trafficking of proteins, such as rhodopsin (*RHO*), guanylyl cyclases (GC), phosphodiesterase (PDE6), is associated with several forms of inherited retinal degeneration (IRD) (3,9–14). Despite our understanding of the components involved in the phototransduction pathway, with the exception of rhodopsin, little is known about the specific mechanisms responsible for the trafficking of proteins to the outer segment where they perform critical functions.

The rod photoreceptor ER is the site for the biogenesis of the integral transmembrane proteins of the OS, such as rhodopsin (12,15). The ER also plays important roles in lipid production and calcium homeostasis. Disturbances in photoreceptor ER function and the induction of ER stress have been associated with several forms of IRD (15–18). Recently, we identified disease-causing mutations in *REEP6*, which encodes the receptor expression enhancing protein 6, in several families with autosomal recessive RP (19). REEP6 belongs to the REEP family of proteins that have been implicated in shaping tubular organelles such as the ER and Golgi, as well as in cellular trafficking of membrane proteins.

We previously used CRISPR/Cas9 gene editing to generate mice with a L135P missense mutation in *Reep6*, which was identified in one individual with recessive RP. The homozygous L135P mice displayed retinal degeneration, including thinning of the outer nuclear layer (ONL) and reduced response to light in dark-adapted animals observed at 4 months of age, confirming potential pathogenicity and the importance of REEP6 for retinal homeostasis (19); however, REEP6 was still detectable at significant levels in the retina of the L135P homozygous mice, and could potentially have some residual activity. Furthermore, most of the variants (4/6) identified in our cohort were predicted to be loss of function mutations with no full-length protein expressed. Therefore, we considered that studying REEP6 null animals, with no residual REEP6 protein or function, could offer unambiguous data on the function of REEP6 and provide insights into the mechanisms of human disease.

In this study, we investigated the detailed phenotype of *Reep6* knockout (KO) mice. *Reep6* knockout (KO) mice exhibit early onset rod dysfunction and photoreceptor degeneration. Using detailed 3D reconstruction, we identified morphological changes in their rod ER and mitochondria. Analysis of phototransduction protein subcellular localization revealed that GC-1 and GC-2 are severely affected in *Reep6* KO mice, suggesting a critical role for REEP6 in their trafficking. We also observed the induction of ER stress, which preceded apoptosis in *Reep6* KO mice. Collectively these data suggest that REEP6 plays a critical role in rod photoreceptor ER homoeostasis and trafficking of essential phototransduction proteins, loss of its function results in impairment of visual transduction processes leading to subsequent retinal degeneration.

## Results

### Generation of *Reep6* mutant mice using CRISPR/Cas9 gene editing

**Figure 1:**
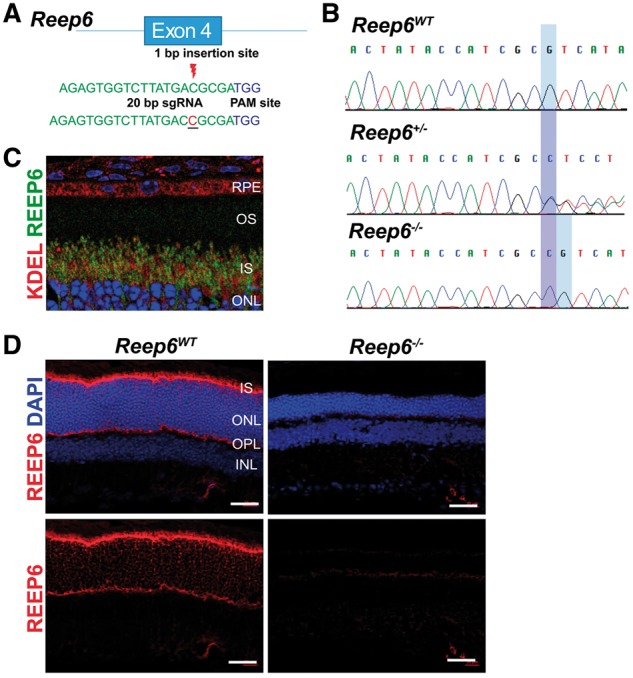
Generation of *Reep6* KO mice. (**A**) Schematic of generation of an insertion mutation in exon 4 of *Reep6* using CRISPR/Cas9 gene editing. (**B**) Genotyping of *Reep6* WT, heterozygous, and homozygous KO mice using Sanger sequencing. (**C**) Expression and localization of REEP6 in mouse retina. REEP6 (green) appears to co-localise with anti-KDEL (red) in the rod photoreceptor inner segment (IS). (**D**) REEP6 (red) expression was restricted to the inner segment (IS) and outer plexiform layer (OPL) of mouse retina shown at P20. No staining was detected in the *Reep6* KO mice.

To elucidate the function of *Reep6*, we generated a KO allele of *Reep6* in mice using CRISPR/Cas9 gene editing. Specifically, exon 4 of *Reep6* was targeted and non-homologous end joining repair was used to create an insertion mutation (Fig. 1A). Using this approach, we generated mice with a 1 bp insertion in exon 4 of *Reep6* that results in a frameshift of the open reading frame (Fig. 1B). Founder mice identified by DNA sequencing were backcrossed to create C57BL/6J congenic lines. *Reep6* KO (*Reep6^-/-^*) mice were viable, fertile, and exhibited no obvious morphological abnormalities upon gross examination.

### *Reep6* deficient mice exhibit progressive photoreceptor degeneration

In the retina, REEP6 expression is detected in the inner segment (IS) of the rod photoreceptor cells and the outer plexiform layer OPL (Fig. 1C) (19,20). REEP6 was not detected in the rod OS or cone photoreceptors in human 3D retinal organoids or in *Reep6* wild-type (WT) mice (19,20). REEP6 expression was undetectable in the retina of the *Reep6* KO mice by immunohistochemistry (Fig. 1D), demonstrating that the CRISPR 1-bp insertion mutation results in a REEP6 deficient mouse. This also confirmed the specificity of the REEP6 antibody.

Immuno-electron microscopy for REEP6 in control WT retina revealed that REEP6 was localized to the ER of the rod inner segment (Supplementary Material, Fig. S1). REEP6 was also occasionally observed close to the sites of contact between the ER and mitochondria, but was not detected within mitochondria (Supplementary Material, Fig. S1).

**Figure 2:**
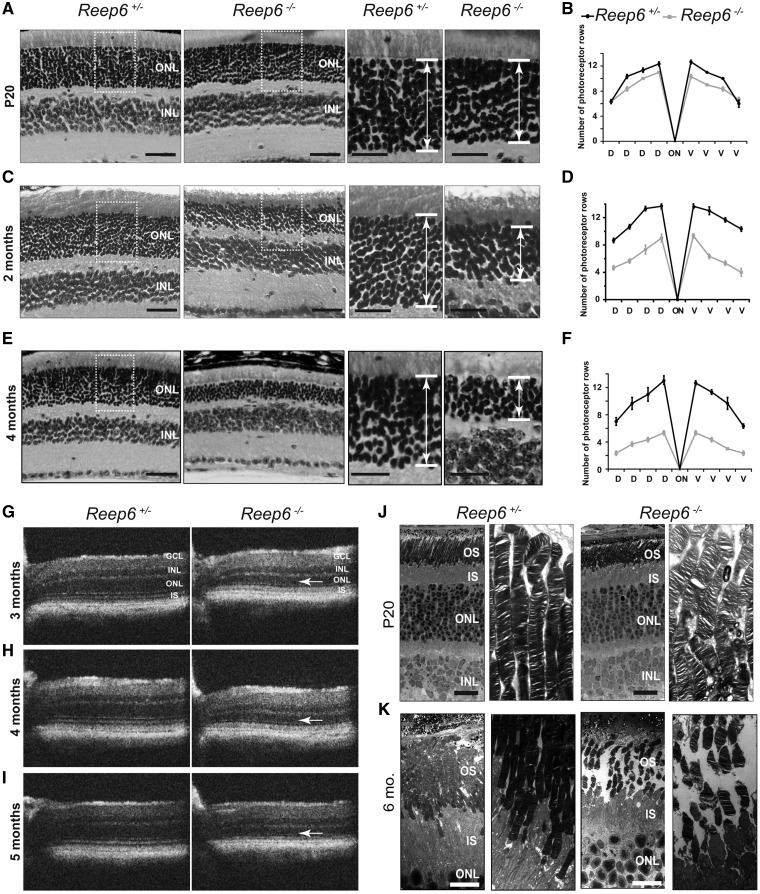
*Reep6* KO mice exhibit photoreceptor degeneration. Histological analysis of retinal sections from *Reep6^+/-^* and *Reep6^-/-^* mice was performed at P20 (**A**) 2 months (**C**) and 4 months (**E**) of age. *Reep6^+/-^* retina shows normal layered organization compared with the *Reep6^-/-^* retina that shows progressive thinning of the ONL. Spidogram of the number of photoreceptor nuclei rows in the ONL of *Reep6^-/-^* mice retinas at P20 (**B**) 2 months (**D**) and 4 months (**F**) of age confirms the progressive loss of photoreceptors is presented as means ±SEM (n = 3). SD-OCT analysis showed thinning of the overall retina progressively from 3 months (**G**), 4 months (**H**) to 5 months (**I**) and thinning of the ONL (arrow) in *Reep6* KO mice compared with *Reep6* heterozygous mice. Transmission EM showing ultrastructure of the photoreceptors at P20 (**J**) and 6 months of age (**K**). ONL, outer nuclear layer; INL, inner nuclear layer; GCL, ganglion cell layer; IS, inner segment; D, dorsal; V, ventral. Scale bars = (A) left: 40 µm, right: 20 µm, (C) left: 40 µm, right: 20 µm, (E) left: 40 µm, right: 20 µm, (J) 2 µm, (K) 20 µm.

Histological analysis of homozygous *Reep6^-/-^* mouse retina revealed defects in retinal morphology compared with the heterozygous *Reep6^+/-^* control retina (Fig. 2). *Reep6* KO mice exhibited mild thinning of the ONL at P20 (Fig. 2A and B), but the severity of the phenotype became more noticeable with age. By 2 months of age, *Reep6^-/-^* mice had degenerated retinas with significant thinning of the ONL and reduced rows of nuclei compared with *Reep6* WT, or heterozygous littermate controls (Fig. 2C and D). By 4 months, only 40% of the ONL rows remained in *Reep6^-/-^*retina (Fig. 2E and F). Furthermore, live retinal imaging by Optical Coherence Tomography (OCT) in adult mice 3 to 5 months old showed progressive thinning of the ONL, consistent with the changes observed histologically (Fig. 2G–I).

To examine the defects at the ultrastructural level, Transmission Electron Microscopy (TEM) on P20 and 6 months old *Reep6^-/-^*, WT and heterozygous *Reep6^+/-^*control mice was performed (Fig. 2J and K). Ultrastructural examination by TEM at P20 revealed no gross abnormalities in retinal structure; however, the stacking of the discs in the outer segment appeared to be slightly disoriented and non-uniform in *Reep6* null mice compared to the heterozygous controls (Fig. 2J). At later stages, a progressive degeneration was observed, evident by greatly reduced and disorganized photoreceptors with shortened, fragmented OS by 6 months of age in *Reep6^-/-^* mice (Fig. 2K), whereas the *Reep6^+/-^*heterozygous retina appeared to be structurally normal.

### *Reep6* KO mice exhibit early-onset physiological changes

**Figure 3:**
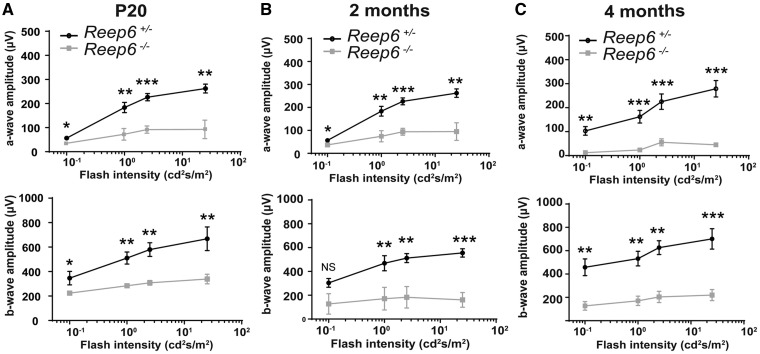
*Reep6^-/-^* mice exhibit defects in response to light. Quantitative evaluation of scotopic a-wave and b-wave for dark-adapted P20 (**A**) 2 month old (**B**) and 4 month old (**C**) *Reep6^+/-^* (black line) and *Reep6^-/-^* mice (grey line). *Reep6^-/-^* mice exhibited decreased ERG responses at all time points. NS, not significant. * Significant at *P*< 0.05. **Significant at *P*< 0.01. ***Significant at *P*< 0.001 determined by Student t-test.

Full-field electroretinography (ERG) of dark-adapted *Reep6^-/-^* mice was performed to evaluate the function of photoreceptors in the retina. *Reep6^-/-^* mice demonstrated a significant reduction in the scotopic response to light as early as P20 compared with heterozygous *Reep6^+/-^* littermate controls (Fig. 3A). Both the a-wave and b-wave amplitudes, measuring the photoreceptor and rod bipolar cells response respectively, were markedly decreased in *Reep6^-/-^* mice, suggesting that the rod photoreception function is significantly compromised by the loss of REEP6. The severely reduced a-wave and b-wave responses were consistent in older *Reep6* KO mice at 2 months and 4 months of age, while the responses were normal in age-matched littermate *Reep6^+/-^*control animals (Fig. 3B and C).

### Loss of Reep6 leads to changes in the rod photoreceptor ER and mitochondria

**Figure 4:**
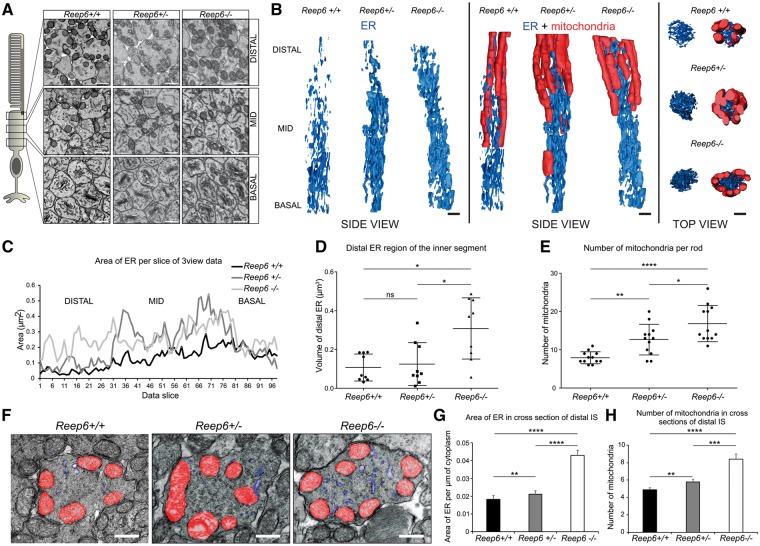
P20 *Reep6* KO mice have an increase in distal rod IS ER volume and proliferation of mitochondria. (**A**) Serial block face SEM images from different regions of rod photoreceptor inner segments (IS) used to generate (**B**) full IS ER and mitochondrial models. Measurements from serial block face SEM data. (**C**) Indicates an increase in ER area in slices within the *Reep6*^-/-^ distal IS, further backed-up by (**D**) measurements from models generated of the distal IS of *Reep6*^+/+^, *Reep6*^+/-^ and *Reep6*^-/-^ rods showing a significant increase in ER volume in *Reep6*^-/-^ (measurements made from models in Supplementary Material, Fig. 2). (**E**) There are more mitochondria in rod IS of *Reep6*^-/-^ compared to *Reep6*^+/-^ and *Reep6*^+/+^ mice. (**F**) TEM images highlighting ER (blue) and mitochondria (red) in sections of the distal portion of *Reep6*^+/+^, *Reep6*^+/-^ and *Reep6*^-/-^ IS. Measurements from TEM images of the distal IS portion of 3 *Reep6*^+/+^, *Reep6*^+/-^ and *Reep6*^-/-^ eyes show (**G**) an increase in ER area and (**H**) mitochondrial proliferation in *Reep6*^-/-^ mice. *Reep6*^-/-^ rods (average = 8) compared to *Reep6*^+/-^ rods (average = 6) and *Reep6*^+/+^ (average = 5) had a significant increase in mitochondrial count. (D) Data are mean ± SD (9 IS). *P < 0.05 determined by Mann-Whitney U test (E) Data are mean ± SD (12 IS). *P < 0.05, **P < 0.01, ****P < 0.0001 determined by Student t-test (G & H) Results are means (three eyes) ± SEM. **P < 0.1, ***P < 0.001, ****P < 0.0001 determined by Student t-test. Scale bars = (A, B) 1µm, (F) 500 nm.

REEP6 is a member of the REEP family of proteins that are related to the Deleted in Polyposis 1 (DP1)/Yop1p family identified in yeast. REEPs and Yop1p are ER resident proteins that act as membrane shaping adaptor proteins to regulate ER membrane structure (21–24). Recent studies have shown that REEP1 is important in neurite development and establishing the structure of the distal ER and in facilitating ER-mitochondria interactions (21,25–27). Therefore, we investigated the structure of the rod photoreceptor ER and mitochondria in *Reep6^-/-^* and littermate control *Reep6^+/-^*mice at P20, before the degeneration was too advanced, by serial block-face scanning electron microscopy (3View) and TEM (Fig. 4A). 3D reconstruction models of the ER of WT (*Reep6^+/+^*), *Reep6^+/-^* and *Reep6^-/-^* rods suggested differences in the organization of the ER at the distal ER close to the base of the outer segment in the *Reep6* KO (Fig. 4B). We developed additional models of the distal region of the ER (Supplementary Material, Fig. S2) and this revealed a significant difference in the area of the ER in this region, with an increase in the total amount of ER near the base of the outer segment (Fig. 4C, D and G). Strikingly, we also observed an increase in the number of mitochondria in the ellipsoid region of the *Reep6^-/-^* rods compared to *Reep6^+/-^* and WT controls (Fig. 4E, F and H). In P20 *Reep6* KO mice, we observed an average of 17 mitochondria (average of 8 in single transverse section) compared with an average of 13 mitochondria (average of 6 in single transverse section) per rod photoreceptor in *Reep6^+/-^* heterozygous mice, and 8 mitochondria (average of 5 in single transverse section) in WT control mice. Collectively, these data suggest that REEP6 is important to establish, or maintain, the architecture of the ER and mitochondria in rod cells.

### The pathological mechanism of REEP6-associated retinopathy

**Figure 5:**
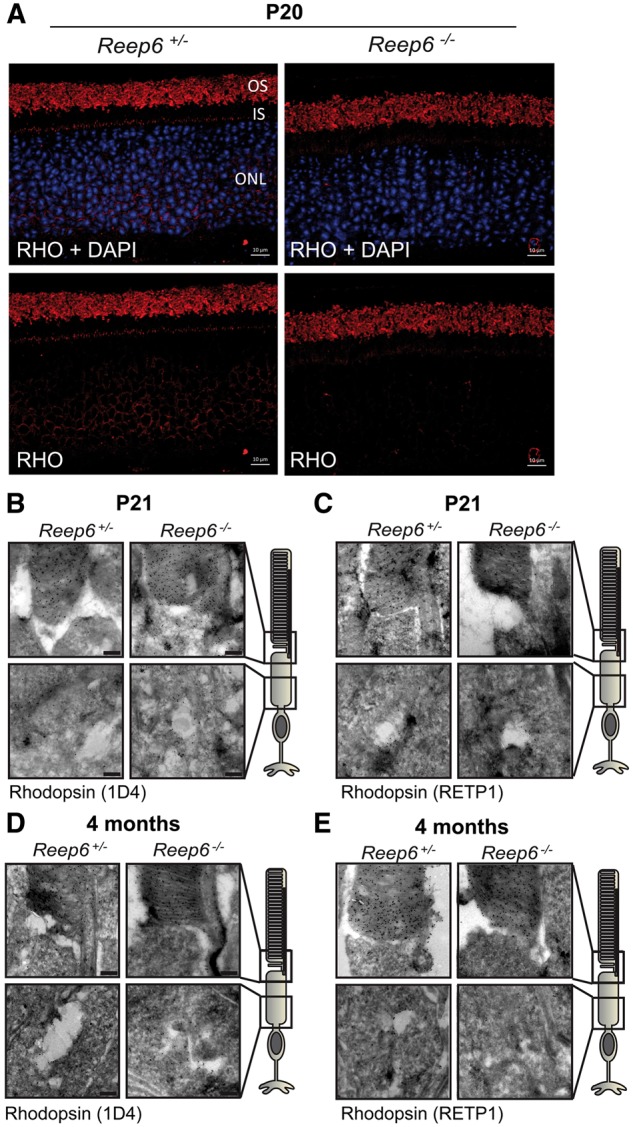
Trafficking of Rhodopsin in *Reep6* KO mice. (**A**) Localization of rhodopsin (Rho; red), is not affected in *Reep6^-/-^* retina at P20. Nuclei are counter-stained with DAPI (blue), in 72 h dark-adapted P20 mice. Immuno-electron microscopy (EM) of rhodopsin using ID4 (**B**) and RETP1 (**C**) in *Reep6* KO mouse retina at P21 reveals no change in localization. (**D** & **E**) Immuno- EM of rhodopsin at 4 months revealed no change in localization. Scale bar= (A) 10 µm, (B, C, D, E) 200nm.

Since the reduction in the ERG responses was observed prior to obvious photoreceptor degeneration, it is likely that the phototransduction pathway is affected in the *Reep6* KO mice. We tested whether the level or subcellular localization of key phototransduction pathway proteins was altered at P20 before degeneration might confound some of the analyses. The six human REEP proteins (REEP1-6) were initially thought to enhance the cell surface traffic of G-protein coupled receptors (GPCR) (Saito et al 2004). Rhodopsin is the archetypal GPCR in rods and the best candidate GPCR to be affected by REEP6; however, rhodopsin protein level and traffic to the outer segment appeared to be unaffected in the *Reep6* null mice compared with the controls (Fig. 5A–E). Since we observed minor defects in the OS disc stacking in *Reep6* KO mice, we also examined the level and localization of the OS structural proteins, retinal outer segment protein 1 (ROM1) and peripherin 2 (PRPH2), which were unaffected (Supplementary Material, Fig. S3), suggesting no major defects in the trafficking of these proteins to the OS.

**Figure 6:**
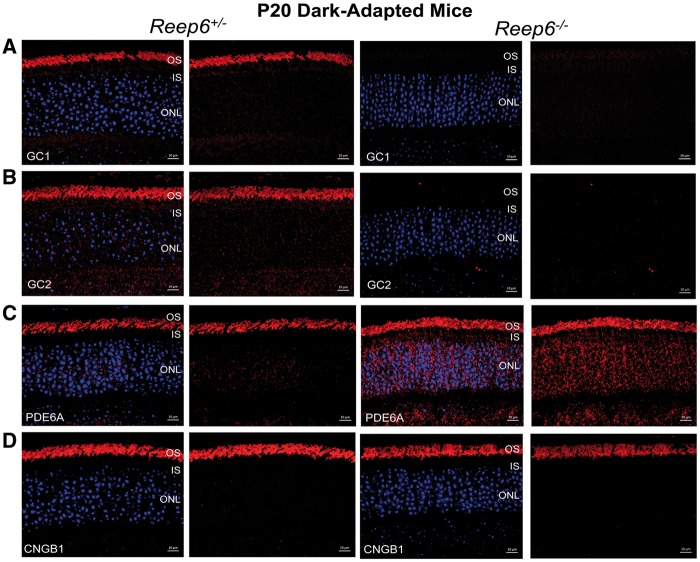
Loss of REEP6 leads to mislocalization of GC1, GC2, and PDE6. (**A**, **B**) Immunofluorescence localization of outer segment proteins GC1 and GC2 (red) is significantly reduced/absent in *Reep6^-/-^* dark-adapted retina at P20 (A, right). (**C**) PDE6A is mislocalized in *Reep6*^-/-^ mouse retina to the ONL and INL regions. (**D**) Localization of CNGB1 (red) is unaffected. Nuclei are counter-stained with DAPI (blue). ONL, outer nuclear layer; INL, inner nuclear layer; IS, inner segment; OS, outer segment. Scale bar= (A–D) 10 µm.

In contrast, defects in the localization of several other members of the phototransduction pathway were observed (Fig. 6). In particular, the localization of guanyl cyclases (GC), enzymes involved in regulating cGMP levels in the photoreceptors, was investigated in *Reep6* null dark-adapted retinas at P20. In *Reep6* null mice, both GC1 and GC2 proteins were undetectable in the OS in comparison to the *Reep6^+/-^* control mice, where they were localized exclusively in the OS as expected (Fig. 6A and B). Furthermore, the localization of PDE6, a central effector of the phototransduction pathway, was observed in the OS in the control retina. In contrast, in the *Reep6* KO retina, increased PDE6 immunoreactivity was detected in the IS (Fig. 6C). Compared to the changes observed in PDE6, GC1 and GC2 were more severely affected. Furthermore, cyclic nucleotide gated channel beta 1 (CNGB1) expression was unaltered in *Reep6* null mice compared to controls (Fig. 6D). Together, these data suggest that loss of REEP6 alters the expression patterns of several integral phototransduction pathway proteins, likely contributing to the photoreceptor dysfunction phenotype observed preceding degeneration.

### ER stress and caspase activation in Reep6 null mice

**Figure 7:**
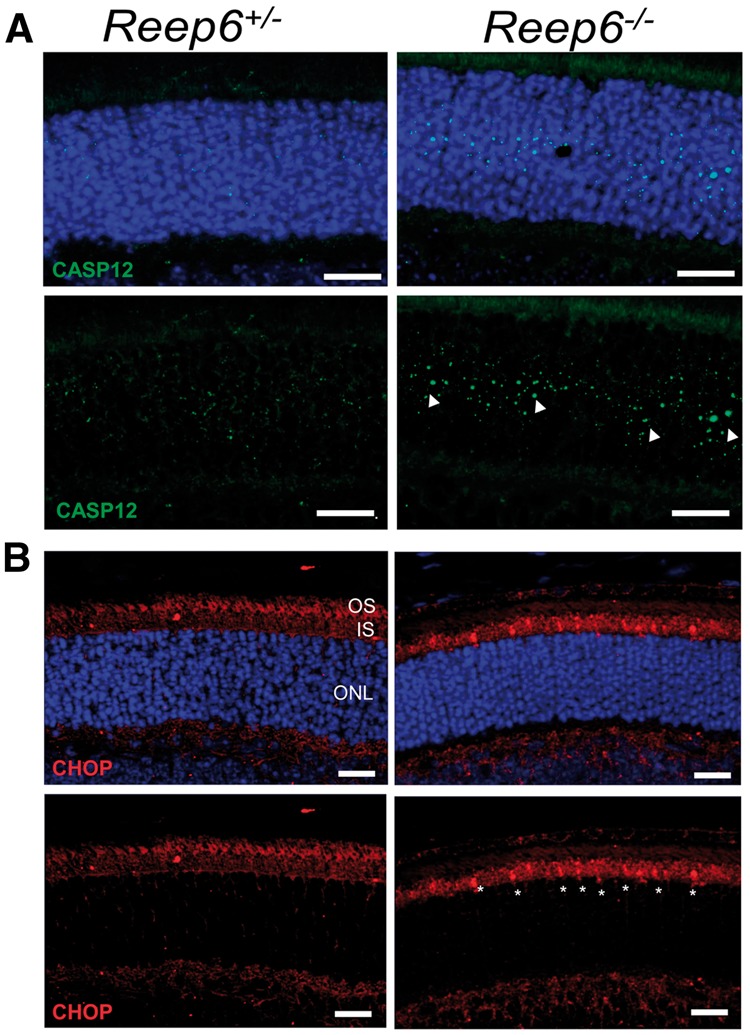
*Reep6^-/-^* mice exhibit ER stress in the retina. (**A**) ER stress was evaluated by immunostaining with ER stress marker Caspase 12 in retinal sections from P20 *Reep6^+/-^* control and *Reep6^-/-^* mice. Caspase 12 (green) positive staining (arrowheads) was observed in the ONL of *Reep6^-/-^* mice retina but absent from the *Reep6^+/-^* control retina. (**B**) CCAAT-enhancer-binding protein homologous protein (CHOP) (red) immunostaining was performed to assay ER stress. CHOP positive punctate staining was observed in *Reep6^-/-^* retina specifically in the IS region of the retina. Nuclei are counter-stained with DAPI (blue). Scale bar= (A & B) 50 µm.

Since *Reep6* KO mice exhibited mistrafficking of phototransduction pathway proteins and alterations in ER structure, we tested whether *Reep6* KO mice exhibit ER stress. The expression of signature ER stress markers was examined in *Reep6* null mice at P20, prior to the development of major phenotypic defects. An increased number of photoreceptor cells were positive for a C/EBP homologous protein (CHOP), which is a downstream pro-apoptotic target of PERK and IRE1, was observed in *Reep6^-/-^* mice compared with the controls. Additionally, the activation of Caspase-12, an ER stress-induced apoptosis marker, was detected in *Reep6^-/-^* mice (Fig. 7A and B), suggesting the activation of the ER stress/unfolded protein response (UPR) pathway.

## Discussion

REEP6 is a transmembrane ER resident protein, and recently we identified mutations in *REEP6* to cause non-syndromic RP in humans (19); however, the function of REEP6 in the retina has previously not been explored. Here, we investigated the phenotype of a REEP6 KO mouse model. The complete loss of REEP6 led to a faster retinal degeneration than we observed in the L135P homozygous knock-in model (19), suggesting that this missense mutation is potentially a hypomorphic allele. The REEP family of proteins has been implicated as membrane-shaping adaptor proteins that are involved in regulating ER sorting and trafficking of G-protein coupled receptors (21). Our findings indicate that REEP6 is required for proper sorting and efficient localization of GC proteins from the ER to the photoreceptor OS. Loss of REEP6 in the retina causes induction of ER stress and defects in the ER and mitochondria organelle homeostasis.

Since the phototransduction pathway begins by capturing photons that are absorbed by rhodopsin, a rod GPCR in the photoreceptors (12,28,29), we examined its expression in *Reep6* KO mice retina to determine whether its trafficking was affected. We found that rhodopsin expression and localization were unaltered in *Reep6* null mice, suggesting that loss of REEP6 does not affect rhodopsin protein trafficking. Photon absorption by rhodopsin leads to activation of the G protein transducin (GαT) (30,31). The alpha- (catalytic) subunit of transducin catalyzes activation of phosphodiesterase (PDE6), which hydrolyzes cyclic GMP (cGMP) to GMP. With decreased cGMP levels, the CNG channels release cGMP and the channel is closed, blocking the flow of calcium and sodium ions into the rod outer segment, causing the photoreceptor to be hyperpolarized (32,33). In dark adapted mice at P20, PDE6 expression was detected in the OS in the control retina, while in the *Reep6* KO retina, PDE6 was mislocalized to the IS and ONL regions.

**Figure 8:**
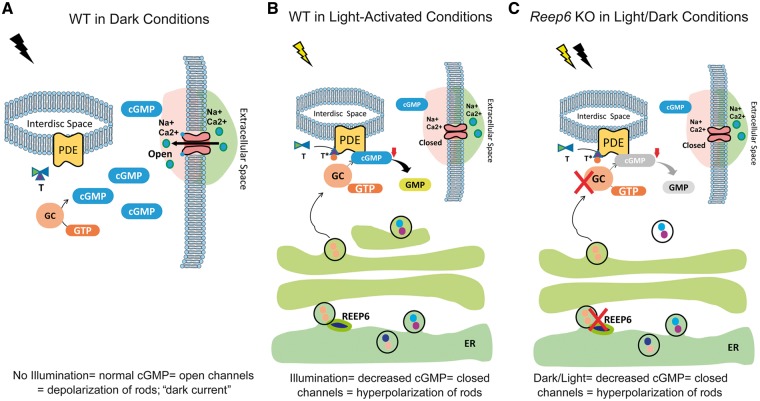
Diagram illustrating the consequences of REEP6 loss in the retina. (**A**) Under normal dark conditions, the phototransduction pathway is not activated by light and the cGMP levels remain basal, the CNG channels are open, and the rods are depolarized. (**B**) In the presence of light, GC converts cGMP to GMP, resulting in decreased cGMP levels, and the CNG channels close. This causes hyperpolarization of the rods. The normal function of REEP6 is to traffic GC from the ER to the OS, where it localizes. (**C**) In the *Reep6* KO mice, trafficking of GC from the ER to the OS is disrupted, resulting in failure of cGMP hydrolysis, and causing failure of CNG channels to open. This leads to prolonged hyperpolarization of the rods photoreceptors.

Furthermore, it is known that cGMP is synthesized by retina-specific guanyl cyclases, called GC1 and GC2, in the OS of photoreceptors (14,34). In the phototransduction cascade, cGMP serves as the second messenger. Activation of the rhodopsin-transducin-PDE6 pathway by photons leads to hydrolysis of cGMP, thereby inducing a conformational switch in the cGMP-gated cation channels in the plasma membrane. In the presence of light, a decrease in the cGMP levels results in closure of the channels and the membrane is in the hyperpolarized state (Fig. 8). GC proteins reset the sensitivity of the rod photoreceptors by restoring the cGMP levels to normal in the dark (30). Conversely, in the dark-adapted retina (in the absence of light), intracellular cGMP does not undergo hydrolysis and thus the ion channels remain open, the rods are depolarized and there is ‘dark current’ present (Fig. 8). Interestingly, we observed that in the absence of REEP6, GC1 and GC2 are dramatically reduced in the OS of the photoreceptor cells in the retina; whereas, in the control P20 retina, robust GC1 and GC2 expression was detected in the OS. This indicates that REEP6 is important for the synthesis, stability and/or trafficking of the cyclases. The absence of GC proteins would lead to a reduction in cGMP in the retina, causing the cGMP gated-ion channels to remain closed (Fig. 8). Thus, irrespective of the light/dark conditions, the rods remain in the hyperpolarized state since GC function is compromised and it fails to replenish the cGMP levels back to normal. This could explain the reduced ERG responses at P20 prior to overt photoreceptor cell loss (Fig. 3). When the rod photoreceptors are irreversibly fixed in the hyperpolarized state, their sensitivity is never reset to normal, likely causing overexertion and subsequent cellular stress. ER and metabolic stress can further explain the abnormal proliferation of mitochondria resulting in their increased density observed in *Reep6* null retina at P20. These factors resulting from loss of REEP6 could cumulatively contribute to the photoreceptor cell death phenotype, delineating the mechanisms of retinal disease pathology in *Reep6* KO mice.

REEP6 appears to play a selective role in the trafficking of OS proteins. Specifically, we find that although rhodopsin is not mislocalized in *Reep6* KO mice, GC1 and GC2 localization is significantly affected. Previous studies have shown that GC1 and rhodopsin co-traffic together, and GC1 is mislocalized in *RHO* KO mice (8). Based on the published reports, it is likely that while rhodopsin is required for proper GC1 trafficking from the Golgi to the photoreceptor OS, rhodopsin trafficking earlier in the secretory pathway could occur independently of GC1 (35). Furthermore, in retinal degeneration 3 (*rd3*) null mice, both GC1 and GC2 are mislocalized and co-immunoprecipitation with GC shows that rd3 interacts with GC (36), indicating that rd3 is required for localization of GCs to the photoreceptor OS. Interestingly, rhodopsin traffics normally to the OS in *rd3* mice, similar to the findings in *Reep6* null mice. Taken together, we suggest that similar to rd3, REEP6 facilitates the synthesis and stability of GC1/2 enabling the exit of vesicles carrying GCs from the ER and their trafficking to the OS of the photoreceptors. The goal of future experiments will be to delineate how REEP6 specifically regulates GC trafficking and whether they interact directly, thereby gaining further mechanistic insights into the underlying functions of REEP6 in intracellular trafficking.

Furthermore, the reduction in GC1 and GC2 in the *Reep6* KO mice could lead to photoreceptor degeneration. Mutations in *GUCY2D* (encoding photoreceptor-specific retinal guanylate cyclase-1) are associated with recessive Leber congenital amaurosis-1 (LCA1) in humans (13,37), and GC1/GC2 double KO (dKO) mice exhibit rapid photoreceptor degeneration, diminished ERG responses, and abnormal OS morphology (38), all phenotypes consistent with the morphological defects observed in *Reep6* KO mice. Additionally, GC dKO mice exhibit low levels of cGMP and decreased rod PDE6 levels (38). In *Reep6* KO mice at P20, we also observed defects in the outer segment and alterations in PDE6 localization, similar to the phenotype in GC dKO mice. Although PDE6 is mistrafficked in *Reep6* KO retina, the effect on GC1 and GC2 levels is more pronounced, suggesting that loss of REEP6 predominantly affects GC1 and GC2 protein trafficking, and that PDE6 is affected downstream of GC trafficking in the OS.

Our study also revealed alteration in the morphology of the ER at P20 when the morphology of the *Reep6* KO retina grossly appears to be minimally affected; however, the ERGs of dark-adapted *Reep6* KO mice show that both the a-wave response and b-wave response are significantly diminished compared with the heterozygous age-matched littermate controls, indicating that the rod photoreceptor cell function is compromised prior to photoreceptor cell death. REEP1 is important in neurite development by establishing the structure of the peripheral ER (26,39) and facilitating ER-mitochondria interactions (27). Interestingly, deletion of *Reep6* affects the peripheral ER of the rod photoreceptor in the region closest to the base of the outer segment. This ellipsoid region of the rod inner segment is also densely packed with mitochondria and deletion of *Reep6* led to an increase in mitochondria in this region, suggesting that REEP6 is important to maintain ER and mitochondrial homeostasis and might also facilitate ER-mitochondria interactions. Nevertheless, it is not clear as yet if this reflects a role for REEP6 in the shaping of the rod peripheral ER, or is a response to cell stress. Previous studies of *Cnga3^-/-^/Nrl^-/-^* mice have shown that defects in cGMP can lead to ER stress (40) and ER stress can lead to changes in ER structure.

Indeed, loss of REEP6 leads to ER stress and induction of the unfolded protein response (UPR) as shown by positive staining for CHOP and Caspase12 in the *Reep6* KO retina at P20. Mutation of ER proteins involved in trafficking and the UPR response are implicated in inherited retinal dystrophies (15,16,41) and ER stress and the UPR have been observed in several types of retinal degeneration (42–44). Our results highlight that loss of REEP6 can also induce ER stress and suggest that induction of the ER stress response pathway genes Capase-12 and CHOP precedes photoreceptor degeneration, implicating ER stress as a possible mechanism for rod cell death in REEP6-RP.

In addition to ER stress, we observed a distinct increase in mitochondrial density in *Reep6* null retina compared with the control retina as assessed by TEM. Mitochondria play a critical role in the retinal cell function and survival, and mitochondrial dysfunction has been observed in several neurodegenerative diseases (45,46). As REEP6.1 appears to be an ER resident protein, it is possible that REEP6 may be involved in regulation of contacts between mitochondria and the ER rather than mitochondrial function and immuno-EM revealed that REEP6 immunoreactivity was also close to potential ER-mitochondrial contact sites (Supplementary Material, Fig. S1), further experiments are required to determine whether the observed mitochondrial proliferation is a direct consequence resulting from loss of REEP6 or a secondary effect adopted by photoreceptors to cope with ER or metabolic/cellular stress inflicted due to loss of REEP6.

In summary, our study elucidates the function of REEP6 in mouse photoreceptors by revealing that REEP6 is involved in production and/or trafficking of selected phototransduction cascade proteins from the ER to the OS. Dysregulation of this process due to loss of REEP6 function results in ER stress and abnormal mitochondrial proliferation, and eventually leads to photoreceptor degeneration. Our findings provide insights into novel homeostatic mechanisms regulated by REEP6, disruption of which leads to ER stress and rod dysfunction, eventually leading to photoreceptor cell death in the retina.

## Materials and Methods

The study protocol received approval from the local ethics committee.

### Generation of *Reep6* KO mice using CRISPR targeting

sgRNA target site for murine *REEP6* was selected using the CRISPR design tool (http://crispr.mit.edu/, AGAGTGGTCTTATGACGCGA**TGG**). As previously described (19), *Reep6* sgRNA was cloned into pDR274 to generate a T7 promoter-mediated sgRNA expression vector. Linearization of the vector was achieved by BsaI digestion. sgRNA was produced using the linearized vector (Maxiscript T7 kit, Life Technologies) and purified with RNA Clean and Concentrator-25 (Zymo Research). Cas mRNA was made as previously described (19). For microinjections to generate *Reep6* K0 mice, Cas mRNA (40 ng/ul) and sgRNA (20 ng/ul) were mixed and microinjected into C57BL/6 embryos at the single-cell stage. To genotype *Reep6* KO mice, we used a genomic PCR assay using the following primers: *Reep6*_F: TCCTGTTCTGGTTCCCTTTCTA and *Reep6* _M13R: CTGCTCAGGAAACAGCTATGACGGAAAAATAAATCCAGCATCCA.

All mice in this study were housed under 12-h light and 12-h dark cycles. All animal operations were approved by the Institutional Animal Care and Use Committee at Baylor College of Medicine.

### Electroretinography

Mice were dark-adapted for 24 h prior to electroretinography (ERG) experimentation. An anaesthesia cocktail (ketamine (22 mg/kg), xylazine (4.4 mg/kg) and acepromazine (0.37 mg/kg)) was delivered by intraperitoneal injection. Under dim red light illumination, each eye was treated with tropicamide (1%) and phenylephrine (2.5%) solutions and the cornea was anesthetized with proparacaine (1%). Goniosoft (2.5%) was applied to the cornea prior to placing ERG electrode on each eye.

A platinum subdermal needle electrode was inserted into the forehead of the mouse as a reference. Scotopic ERG was performed at six flash intensities (−24, −14, −4, 0 and 10 dB) as previously described (47) on post-natal day 20 (P20) to 4 months of age for *Reep6* KO and littermate control mice. The LKC UTAS Visual Diagnostic System and EMWIN software (LKC Technologies, Gaithersburg, MD) was utilized to digitize and store the recordings. A-waves (photoreceptor response) were measured from baseline to the negative deflection trough, and b-waves (on bi-polar cell response) were measured from the a-wave trough to the positive deflection peak. All ERG data were analysed and plotted using GraphPad Prism5 software (GraphPad Software, La Jolla, CA, USA).

### Spectral-domain optical coherence tomography

Ultra high resolution Spectral Domain Ophthalmic Imaging System (SD-OCT) (Envisu R2200 SDOIS, Leica Microsystems, Morrisville, NC) was used to perform live-imaging of mouse retinas. Prior to imaging, intraperitoneal injection of a ketamine/xylazine anaesthesia cocktail (ketamine, 100 mg/kg; xylazine, 10 mg/kg) followed by dilation of eyes with one drop of 1% cyclopentolate hydrochloride ophthalmic solution (Baush & Lomb, Rochester, NY) followed by one drop of 2.5% phenylephrine hydrochloride ophthalmic solution (Falcon Pharmaceuticals, Fort Worth, TX) was performed. Pre-dilation, eyes were lubricated using Systane Ultra lubricant drops (Alcon, Fort Worth, TX) and GenTeal Severe lubricant gel (Alcon, Fort Worth, TX) was used post-dilation. Mice were placed in the cylindrical mouse cassette on the rodent alignment stage (RAS) in front of the optical scanning mouse retina bore. Pupils were adjusted as needed and each eye was imaged along the entire axial length. Image acquisition, assessment and processing were performed using the InVivoVue 2.2 Image Acquisition Software (Leica Microsystems).

### Histology and immunostaining

Mouse eyes were dissected fixed in freshly prepared Modified Davidson's Fixative (48) overnight at 4 °C. Fixed eyes were processed through ethanol dehydration series (50, 70, 95, and 100%) for 1 h each, followed by paraffin-embedding. Serial paraffin sections (7 µm) were obtained (Microtome, Leica) and H&E stained according a standard protocol. All H&E stained slides were visualized using light microscopy (Zeiss Apotome). For immunohistological staining, paraffin tissue section slides were dewaxed in xylene for 1 h followed by alcohol rehydration series. Antigen retrieval was performed by boiling the slides in 0.01M citrate buffer for 30 min. Slides were then cooled and hybridized (10% normal goat serum, 0.1% Triton-X 100, PBS) for 1 h before incubation with the following primary antibodies: REEP6 (Proteintech 12088-1-AP), REEP6 (kind gift of Anand Swaroop, NEI), Rhodopsin (sc-57432), Caspase 12 (EMD Millipore, ab3612), CHOP(sc-575), KDEL (10C3, EMD Millipore, 420400), Rom1 and RDS (kind gift of R.S. Molday, UBC). Slides were washed 5 times in PBS and incubated with secondary antibodies (Molecular Probes) for 1 h. After three PBS rinses, DAPI was used to counterstain the nuclei. Slides were rinsed again in PBS and mounted with ProLong Gold antifade reagent (Life Technologies). Images were obtained using a fluorescence microscope (Zeiss Axio Imager M2m).

### Transmission electron microscopy

Fresh eye cups were dissected from mice and fixed in 2.5% glutaraldehyde, 1% paraformaldehyde and 0.1M Phosphate buffer pH 7.3 at  4 °C for 48 h. Tissues were post-fixed in 1% osmium tetroxide/1N sodium cacodylate buffer for 2 h and dehydrated in a series of ethanol gradients. Conjunctiva samples were infiltrated with acetone and PolyBed 812 plastic resin and embedded in plastic molds with 100% plastic resin. Sections of 1 micron (thick) and 80–90 nm (thin) were cut using Leica Ultracut R ultramicrotome. Thick sections were stained with Toluidine Blue stain and ultrathin sections were placed on copper mesh grid stained with uranyl acetate and lead citrate. A Zeiss EM902 and a JEOL 1010 were used for visualizing the sections and imaging was performed on AMT V602 and SC1000B digital camera respectively. Measurements of the ER and mitochondria were performed using ImageJ (NIH) and analysed in Prism (Graphpad).

### Cryo-immuno-electron microscopy

Eyes were fixed in 4% paraformaldehyde and 0.1% glutaraldehyde in 0.1M phosphate buffer pH 7.4. Small blocks of tissue were prepared from the eyes and embedded in 12% gelatin before infusing with 2.3 M sucrose solution overnight at 4 °C. 100 nm sections were cut at −120 °C and collected on electron microscopy grids in 1:1 mixture of 2.3M sucrose/2% methylcellulose. Immuno-labelling was performed as described previously (49). The following antibodies were used: Drp1 (BD Bioscience, 611113), TOM20 (sc-17764), REEP6 (Proteintech 12088-1-AP), Rhodopsin 1D4 (Abcam-ab5417) and Rhodopsin RETP1 (Abcam-ab3267). Images were acquired on a JEOL 1010 transmission electron microscope equipped with a Gatan Orius SC1000B charge-coupled device camera.

### 3View serial block-face scanning electron microscopy

Mouse eyes fixed in 3% glutaraldehyde and 1% paraformaldehyde in 0.08 M sodium cacodylate buffer, pH 7.4, were sequentially en bloc stained with 1% osmium 1.5% potassium ferricyanide in 0.08M cacodylate buffer, 1% aqueous sodium thiocarbohydrazide, 1% aqueous osmium, 1% aqueous uranyl acetate followed by Walton’s lead aspartate (50). The samples were dehydrated using a series of increasing concentrations of ethanol (50, 70, 90, 3x 100%) followed by propylene oxide and infiltration in a mixture of propylene oxide and Durcupan ACM resin (1:1), before embedding in Durcupan ACM resin. Blocks cut from the embedded specimens were superglued onto aluminum pins prior to coating with gold palladium. Images were acquired in between sequential sectioning (100nm thick) of the block surface using a Gatan 3View system (Gatan Inc, Abingdon, UK) and a Zeiss Sigma VP field emission scanning electron microscope (Zeiss, Cambridge, UK). The images were re-aligned using the StackReg plugin (EPFL) in ImageJ (NIH) and modelling was performed using Amira 5.3.3 software (FEI). Measurements of photoreceptor inner segment ER and mitochondria of were made using 9 and 12 rods respectively from *Reep6* KO and heterozygous mice in Amira and analyzed in Prism (Graphpad).

## Supplementary Material

Supplementary Data
