# Supplementary material for: REEP6 deficiency leads to retinal degeneration through disruption of ER homeostasis and protein trafficking
Source: Hum Mol Genet. 2017 May 5;26(14):2667–77. doi: 10.1093/hmg/ddx149 (PMC5808736; doi:10.1093/hmg/ddx149)
Supplement: Supplementary Data [file ddx149_supp.pdf]

**Supplementary Information**

# REEP6 Deficiency Leads to Retinal Degeneration through Disruption of ER Homeostasis and Protein Trafficking

Smriti A. Agrawal,[1,2] Thomas Burgoyne,[3] Aiden Eblimit,[1,2] James Bellingham,[3] David A. Parfitt,[3] Amelia Lane,[3] Ralph Nichols,[4] Chinwe Asomugha,[5] Matthew J. Hayes,[3] Peter M. Munro,[3] Mingchu Xu,[1,2] Keqing Wang,[1,2] Clare E. Futter,[3] Yumei Li,[1,2] Rui Chen[1,2]* and Michael E. Cheetham,[3]*

[1] Department of Molecular and Human Genetics, Baylor College of Medicine, Houston, TX 77030-3411, USA;

[2] Human Genome Sequencing Center, Baylor College of Medicine, Houston, TX 77030-3411, USA;

[3] UCL Institute of Ophthalmology, 11-43 Bath Street, London EC1V 9EL, UK;

[4] Department of Ophthalmology, Baylor College of Medicine, Houston, TX 77030-3411, USA;

[5] Department of Molecular Physiology and Biophysics, Baylor College of Medicine, Houston, TX 77030-3411, USA;

***Corresponding author information:**

Rui Chen, ruichen@bcm.edu, Phone: +1- 713-798-5194, Fax: +1- 713-798-5741

Mike Cheetham, michael.cheetham@ucl.ac.uk, Phone: +44 20 7608 6944, Fax: +44 20 7608 6892

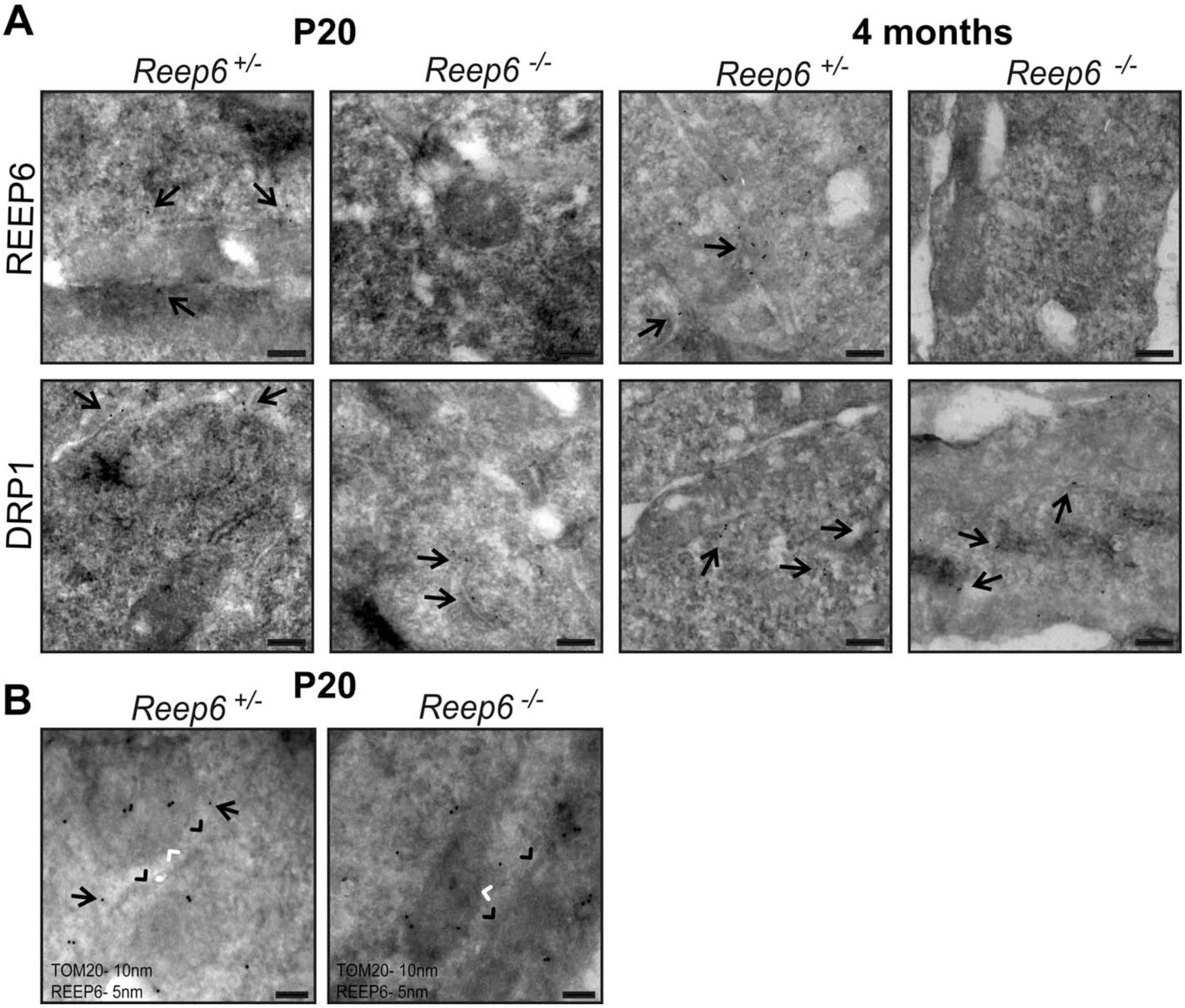


**Supplemental material Fig.1** Immuno-EM labelling of REEP6, Drp1 and Tom20. REEP6 immunoreactivity is detected close to potential ER-mitochondrial contact sites. Immuno-EM using TOM20, a mitochondrial marker and Drp1 (dynamin-related protein 1), a mitochondrial-ER interface marker, shows that REEP6 is expressed near the ER-mitochondrial contact sites. (**A**) Arrows indicate immuno-gold labelling of REEP6 and Drp1. (**B**) Dual immuno-EM labelling of Tom20 (10nm gold) and REEP6 (5nm gold). Arrows highlight REEP6 labelling, black arrow heads ER and white arrow mitochondria at sites of potential mitochondrial-ER interface. Scale bar= (**A**) 200nm, (**B**) 100nm.

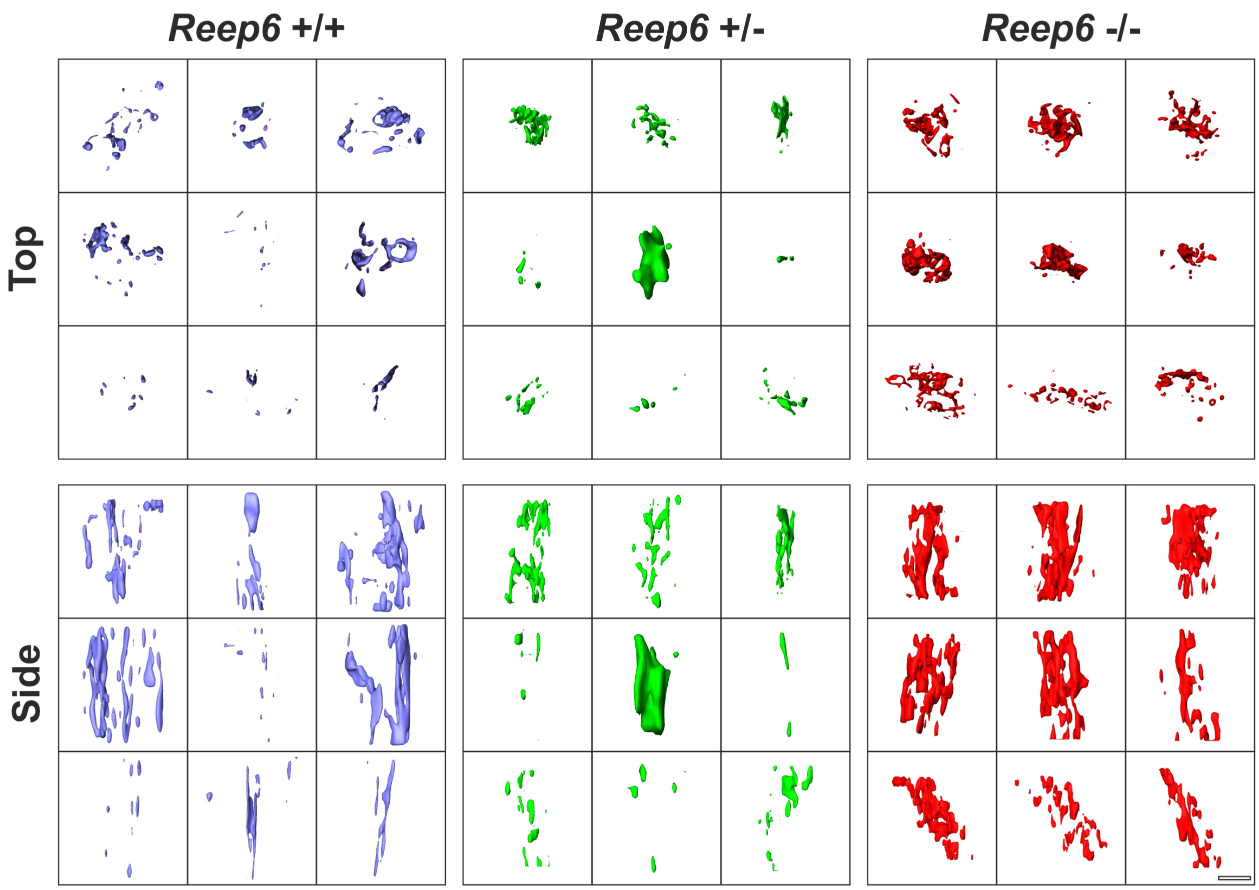


**Supplemental material Fig. 2** Models of distal inner segment (IS) ER generated from the serial block-face SEM data of nine *Reep6*$^{+/-}$, *Reep6*$^{+/-}$ and *Reep6*$^{-/-}$ photoreceptors at P20. Top view and corresponding side view of models used to calculate the volume of distal IS ER. Scale bar= 200nm

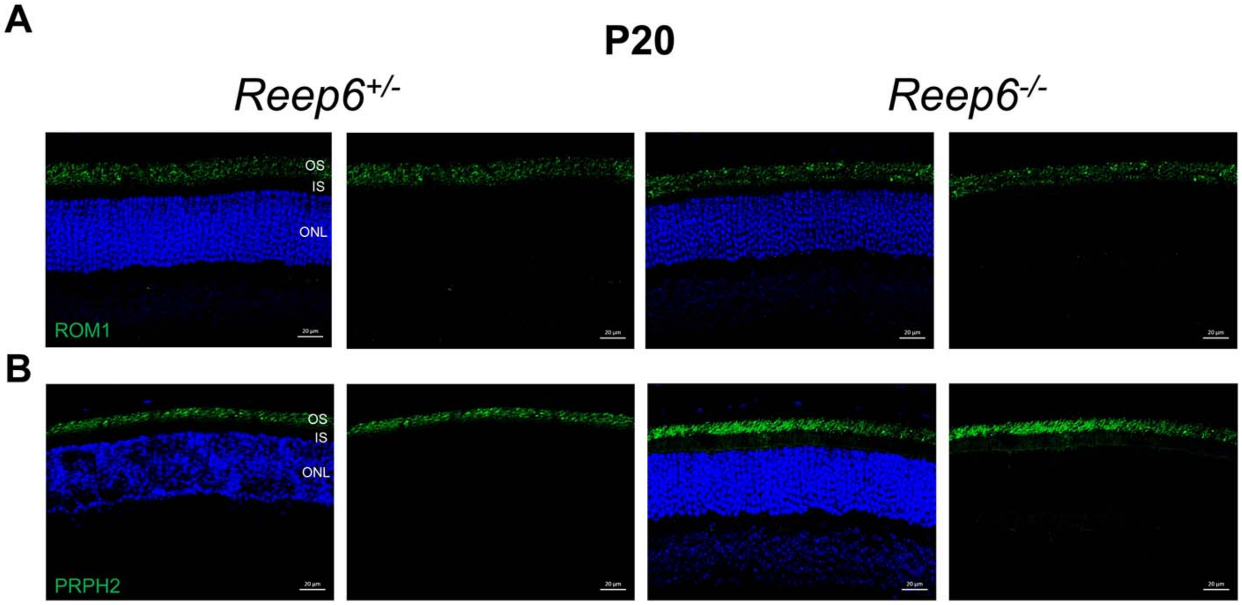


**Supplemental material Fig. 3** Trafficking of ROM1 and PRPH2 is unaffected in *Reep6* KO mice at P20. (**A**) Localization of retinal outer segment membrane protein 1(ROM1), shown in green, is not affected in *Reep6*$^{-/-}$ retina at P20. (**B**) Localization of disk morphogenesis protein, peripherin 2 (also known as known as RDS), is not affected in *Reep6*$^{-/-}$ retina at P20. Nuclei are counter-stained with DAPI (blue). Scale bar= (**A**, **B**) 20 μm.
